# Extraordinarily large swelling energy of iodine-treated poly(vinyl alcohol) demonstrated by jump of a film

**DOI:** 10.1002/polb.23570

**Published:** 2014-08-18

**Authors:** Tatsuro Takamura, Kazuya Nozawa, Yoshiki Sugimoto, Masatoshi Shioya

**Affiliations:** 1 Tokyo Research Laboratory, Mitsubishi Gas Chemical Company Inc., 1-1 Niijuku 6-ChomeKatsushika-Ku, Tokyo, 125-8601, Japan; 2 Department of Organic and Polymeric Materials, Tokyo Institute of Technology, 2–12-1-S8–34 O-OkayamaMeguro-Ku, Tokyo, 152-8552, Japan; 3 Department of Technology Management for Innovation, School of Engineering, University of Tokyo7-3-1 Hongo, Bunkyo-Ku, Tokyo, 113-8656, Japan

**Keywords:** actuators, fibers, films, mechanical properties, poly(vinyl alcohol), swelling

## Abstract

Organic material characteristics of volume change and stress generation have attracted the attention of many researchers aiming to develop chemomechanical systems such as artificial muscles and polymer engines having the advantages of high energy density and silent operation. Although polymer gels offer a relatively large actuator stroke, their mechanical properties are relatively poor and the working temperature is relatively low, often limited by the evaporation of liquid if contained. We have developed an iodine-treated poly(vinyl alcohol) having extraordinarily large vapor-induced deswelling stress reaching 59 MPa, which is one to two orders of magnitude greater than those of ordinary polymer gels. Furthermore, this material has extremely large volumetric and gravimetric energy densities reaching 1.3 × 10^6^ J m^−3^ and 9.6 × 10^2^ J kg^−1^, respectively, and an elastic modulus of a few GPa and is heat-resistant to at least 200 °C. The high performance of this material can be demonstrated by a jump of a film. © 2014 The Authors. Journal of Polymer Science Part B: Polymer Physics published by Wiley Periodicals, Inc. J. Polym. Sci., Part B: Polym. Phys. **2014**, *52*, 1357–1365

## Introduction

Many types of organic materials have been developed which experience volume change and generate stress in response to various stimuli such as light, electric field, heat, pH, and chemical substances.^1^–^11^ Among various mechanisms of volume change, swelling of polymer gels is caused by a mechanism similar to that responsible for dissolution of polymers in solvents. Although the crosslinks between polymer chains in gels prevent solution-like flow, swelling significantly reduces the rigidity of ordinary polymer gels. We have developed a crosslinked structure that can prevent this significant loss in rigidity without sacrificing swelling ability by an iodine treatment of poly(vinyl alcohol) (PVA).

Iodine doping of PVA has been used for producing polarizers, through which a complex of polyiodide and PVA molecules is formed.^12^ Iodine doping is also known to enhance the drawability of PVA due to the reduction of intermolecular interactions.^13^ When compared with these iodine treatments, the iodine treatment we applied is very intensive: the starting PVA was exposed to iodine vapor at 100 °C for 24 h. Similar treatment can be applied in the process of producing carbon materials from PVA because this treatment effectively improves the yield of carbon if applied prior to carbonization at a high temperature.^14^,^15^

The iodine-treated PVA film produced in this manner jumps on a plate wiped with a solvent-wetted cloth, which demonstrates the high performance of this material. In this study, jumping motion of the film has been analyzed in connection with the mechanical, thermal, and swelling properties of this material. The mechanism for producing extremely large swelling energy has also been discussed.

## Experimental

### Materials

The starting PVA films were prepared by dissolving a PVA powder (5 g) with a polymerization degree of 900–1100 and a saponification degree of 86–90% (Wako Pure Chemistry Industries) in distilled water (50 mL) at 90 °C, casting the solution and drying the films at room temperature for 48 h. Two types of fibers spun from PVA with a polymerization degree of 1700 and a saponification degree of 99.9% and differing in diameter were used as the starting PVA fibers.

### Iodine Treatment

The starting PVA films and fibers were exposed to saturated iodine vapor by placing them into a glass vessel together with an iodine powder with a purity of 99.8% (Wako Pure Chemistry Industries), depressurizing the vessel for 30 min and heating the sealed vessel at 100 °C for 24 h. To remove iodine deposited on the surface, the iodine-treated PVA films and fibers were taken out from the vessel after depressurization at 100 °C for 1 h and washed in methanol at 40 °C for 24 h. During the iodine treatment, the films were placed on a rough grid made of thread so that both sides of the films were exposed to the iodine vapor. By contrast, the fibers were under tension at a stress of 5.4 MPa unless otherwise noted to prevent shrinkage. The iodine-treated PVA films had thicknesses ranging from 115 to 138 μm. The diameter of the iodine-treated PVA fibers differed between the two starting PAN fibers and further varied depending on the tension applied during the iodine treatment.

### Bending Test

The elastic modulus (which corresponds to the Young modulus determined by the tensile test but is different from the bending rigidity in definition) of the films was determined by performing the three-point bending test using an universal material testing instrument (Tensilon RTC-1350A; A&D) The elastic modulus, *E*, was determined using the following equation:


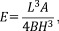
(1)

where *L* is the span length, *H* is the specimen thickness, *B* is the specimen width, and *A* is the ratio of the bending load against the bending deflection.

The changes in the elastic modulus with time (relaxation modulus) were measured by holding a constant bending deflection and measuring the changes in the bending load with time. The films 30 mm long and 8 mm wide were tested at a span length of 20 mm. A bending deflection of 5 mm was initially imposed at a crosshead speed of 30 mm min^−1^ and held during the measurement.

The changes in the elastic modulus with temperature were measured by imposing the bending deflection repeatedly at a constant crosshead speed at various temperatures. As the value of *A* in eq 1, the initial slope of the bending load–bending deflection curve was used. The films 30 mm long and 8 mm wide were tested at the span length of 18 mm and the crosshead speed of 5 mm min^−1^. The crosshead speed to impose bending deflection influences the elastic modulus. A higher crosshead speed, however, was used for the measurements of time evolution when compared with the measurements of temperature dependence to reduce relaxation when applying the bending deflection.

### Measurements of Swelling Property

**Figure 1:**
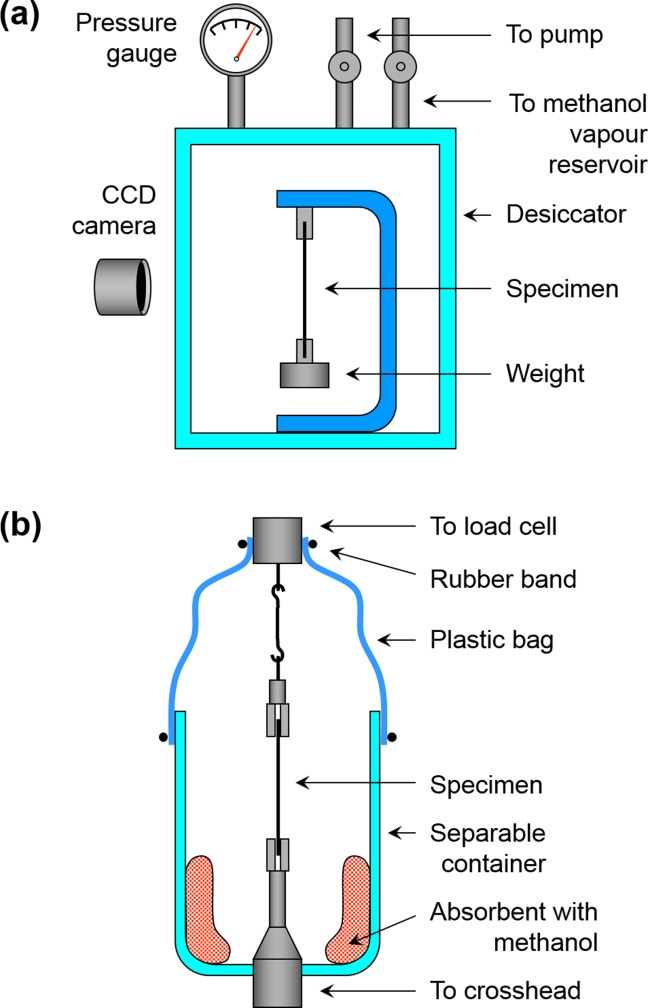
Experimental setup for measuring (a) swelling strain and (b) contraction stress of fibers. [Color figure can be viewed in the online issue, which is available at http://wileyonlinelibrary.com.]

Changes in the fiber length when exposed to methanol vapor were measured by hanging the fiber vertically in a desiccator [Fig. 1(a)]. During the measurement, the fiber was straightened by hanging a weight corresponding to a tensile stress of 2.6 or 5.0 MPa. After evacuating the desiccator, methanol vapor was introduced into the desiccator. The changes in the fiber length were analyzed in photographs of the fibers taken with a CCD camera. The increase in fiber length was caused by not only swelling itself but also a reduction in the elastic modulus. However, as the reduction in the elastic modulus of the iodine-treated fiber was from 2.4 GPa in a dry state to 1.5 GPa in methanol vapor at 12 kPa, the fiber strain caused by the reduction in elastic modulus was only about 0.1% at maximum.

The contraction stress of the fiber due to deswelling (deswelling stress) was measured by using the universal material testing instrument and, for sealing the vapor, a container which can be easily taken off from the testing instrument [Fig. 1(b)]. The methanol vapor was supplied from the methanol-wetted absorbent in the container. When the fiber reached in equilibrium with methanol vapor, the fiber was straightened by adjusting the position of the crosshead of the testing instrument, and the container was quickly taken off from the testing instrument for removing methanol vapor from the atmosphere. The contraction stress of the fiber was measured at a constant fiber length.

## Results and Discussion

### Chemical and Structural Changes

**Figure 2:**
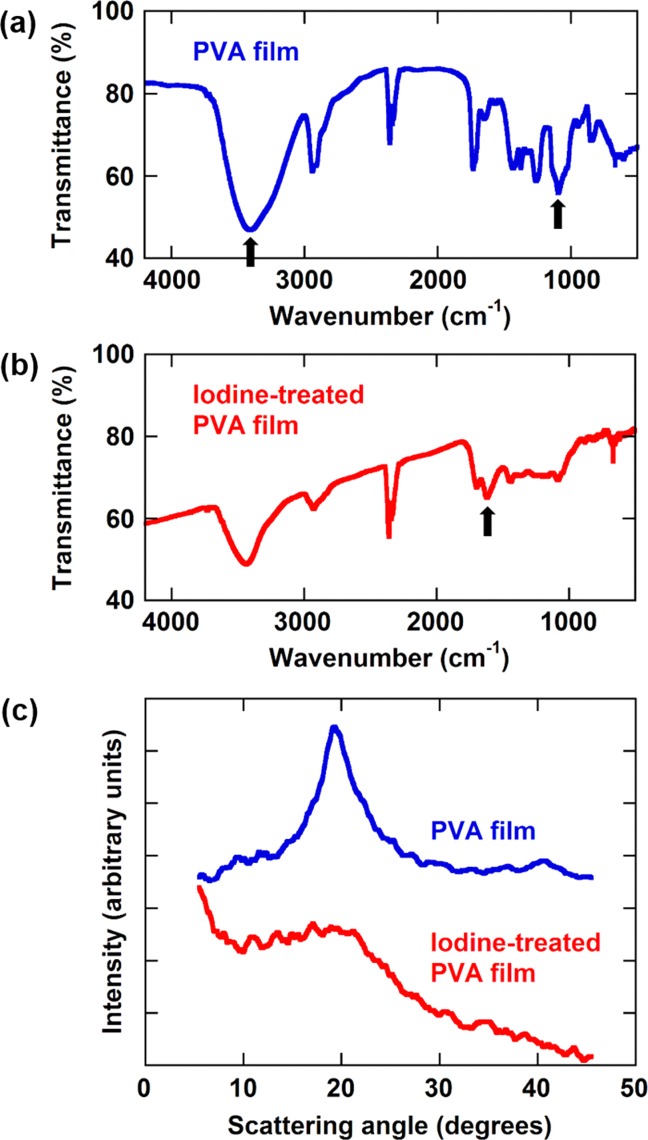
IR spectra of (a) starting PVA film and (b) iodine-treated PVA film. (c) WAXD profiles of starting PVA film and iodine-treated PVA film measured using Cu Kα X-ray. [Color figure can be viewed in the online issue, which is available at http://wileyonlinelibrary.com.]

The IR absorption spectra of the starting PVA film and the iodine-treated PVA film are shown in Figure 2(a,b). The O—H stretching vibration at about 3400 cm^−1^ and the C—O stretching vibration at 1083–1090 cm^−1^ [indicated by the arrows in Fig. 2(a)] were reduced due to dehydration by the iodine treatment. The development of C—C stretching vibration at about 1621 cm^−1^ [indicated by the arrow in Fig. 2(b)] suggests that polyene was formed by the iodine treatment. It has been reported that the iodine treatment of PVA in combination with heat treatment produces polyene due to dehydration and then aromatic rings and crosslinks due to intermolecular ring closure of polyene.^16^ The mass fraction of iodine remaining in the iodine-treated PVA was estimated to be ∼20% using a reported method.^14^,^17^

The WAXD profiles of the starting PVA film and the iodine-treated PVA film are compared in Figure 2(c). The iodine treatment caused significant reduction in (101) reflection^18^ due to the destruction of monoclinic PVA crystallites.

### Mechanical and Thermal Properties

**Figure 3:**
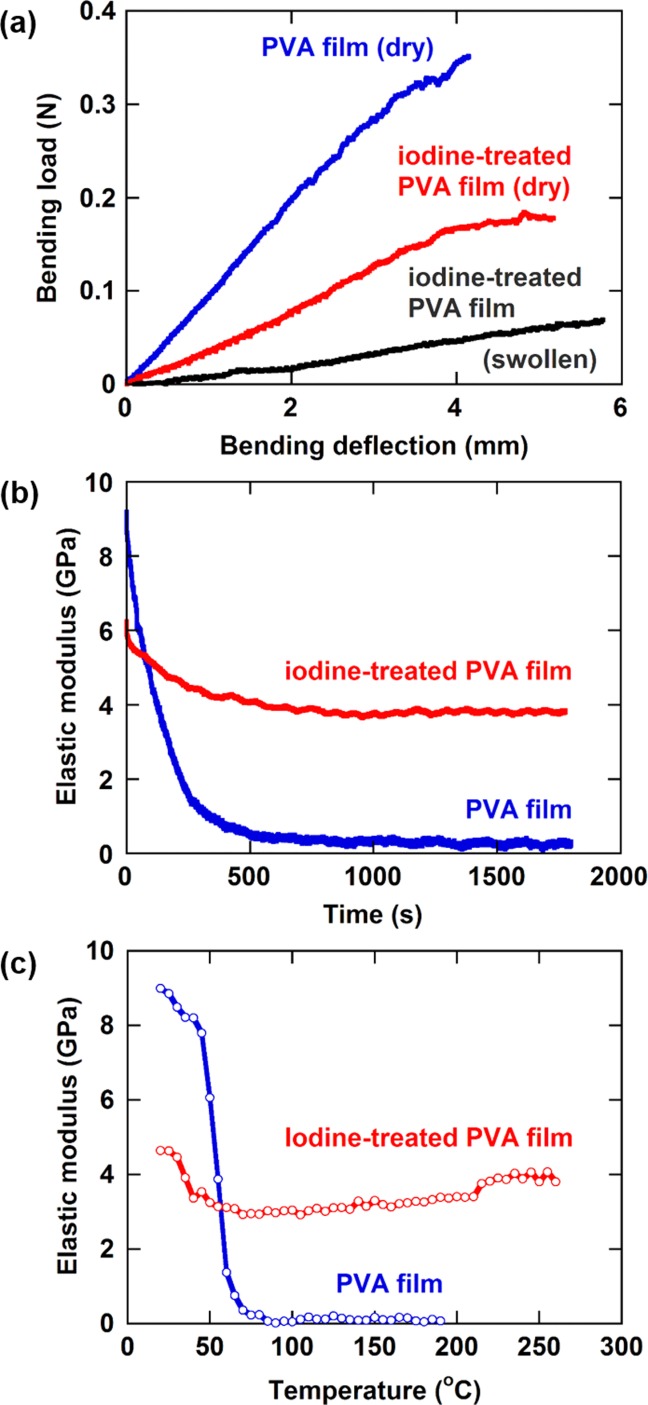
Mechanical properties of starting PVA film and iodine-treated PVA film. (a) Bending load–bending deflection curves of three-point bending tests at room temperature on the films in a dry state and a swollen state after soaked in methanol. The starting PVA film became too soft for a bending test to be conducted when soaked in liquid methanol. (b) Changes in the elastic modulus with time measured at a constant bending deflection at 45 °C. (c) Changes in the elastic modulus with temperature. [Color figure can be viewed in the online issue, which is available at http://wileyonlinelibrary.com.]

Figure 3(a) compares the bending load–bending deflection curves of the three-point bending tests at room temperature on the starting PVA film and the iodine-treated PVA film in a dry state and a swollen state after soaked in liquid methanol. The starting PVA film became too soft for a bending test to be conducted when soaked in liquid methanol.

Polymers show viscoelastic mechanical response to a greater or lesser extent, and the relative strengths of the viscous and the elastic character can be represented by a relaxation time. A material with a shorter relaxation time shows a more viscous response: it shows faster decays in the stress and elastic modulus when held at a constant deformation (stress relaxation) and the larger energy dissipation when stretched at a constant rate. Figure 3(b) compares changes in the elastic modulus with time for the starting PVA film and the iodine-treated PVA film at 45 °C. The smaller initial elastic modulus for the iodine-treated PVA film relative to the starting PVA film was due to the destruction of crystallites by the iodine treatment. The decrease in the elastic modulus with time, however, was significantly suppressed for the iodine-treated PVA film. The elastic modulus of the iodine-treated PVA film approached asymptotically to a constant value, which indicates that this material has an extremely long relaxation time component in the relaxation time spectrum.

Figure 3(c) compares the changes in the elastic modulus with temperature for the starting PVA film and the iodine-treated PVA film. Although iodine treatment reduced elastic modulus, it prevented a significant reduction in the elastic modulus at elevated temperatures. For the starting PVA film, a significant reduction in the elastic modulus occurs at the glass transition temperature of ∼40 °C. For the iodine-treated PVA film, the reduction in elastic modulus is greatly suppressed although it occurs at about 40 °C due to a remaining PVA domain. The elastic modulus of this film was kept at no less than 60% of the value at room temperature at least up to 260 °C. A gradual increase in the elastic modulus of this film beyond 60 °C was attributed to the progress of dehydration reaction caused by the residual iodine.

**Figure 4:**
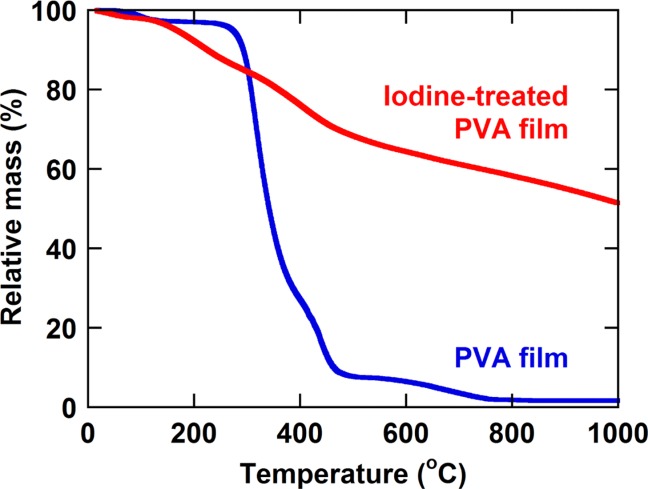
Thermogravimetric analysis curves of starting PVA film and iodine-treated PVA film measured under nitrogen gas flow at a heating rate of 10 °C min^−1^. [Color figure can be viewed in the online issue, which is available at http://wileyonlinelibrary.com.]

Figure 4 compares the thermogravimetric analysis curves of the starting PVA film and the iodine-treated PVA film. The starting PVA film showed weight losses at about 100 °C due to evaporation of water and about 300 °C due to decomposition of PVA. The iodine-treated PVA film showed weight losses at about 120 °C due to sublimation of remaining iodine and at higher temperatures due to the progress of dehydration reaction. However, the weight loss was greatly suppressed relative to that of the starting PVA film. Under ordinary conditions, the iodine-treated PVA was stable for a long period of time.

### Swelling Properties

**Figure 5:**
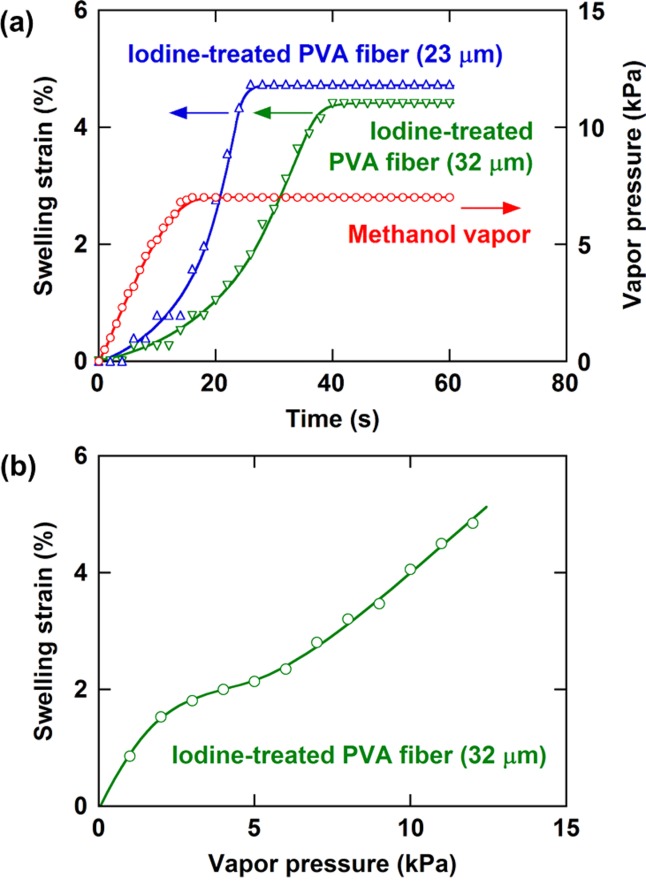
Swelling behavior of iodine-treated PVA fibers with the indicated diameters. (a) Changes in the swelling strain of the fiber and the methanol vapor pressure with time after methanol vapor were introduced in the desiccator containing the fiber at 20 °C. (b) Swelling strain of the fiber in equilibrium with methanol vapor at various pressures. [Color figure can be viewed in the online issue, which is available at http://wileyonlinelibrary.com.]

It is considered that the difference in the thickness between the starting PVA films and fibers influences the iodine concentration inside the material during iodine treatment and the swelling properties of the resulting iodine-treated PVA films and fibers. The fibers, however, were used for measuring the ability of this material to generate strain and stress by swelling and deswelling as fibers are advantageous when compared with films in reducing ununiformity of the degree of swelling in the specimen. Figure 5(a) shows the variation of the swelling strain of the iodine-treated PVA fibers after exposure to methanol vapor, where the swelling strain is defined as the relative increase in fiber length when compared with the initial fiber length. Following the rapid increase in the methanol vapor pressure, the swelling strain of the fibers increased with a certain time lag and reached 4.7% and 4.4% for the 23- and 32-μm diameter fibers, respectively. The response time calculated from the time difference between when the vapor pressure and the fiber length reached constant values was ∼10 and 24 s for the 23- and 32-μm diameter fibers, respectively. The larger surface area-to-volume ratio of the thinner fiber contributed to the faster methanol uptake. The swelling strain of the fiber in equilibrium with methanol vapor at various pressures is shown in Figure 5(b).

**Figure 6:**
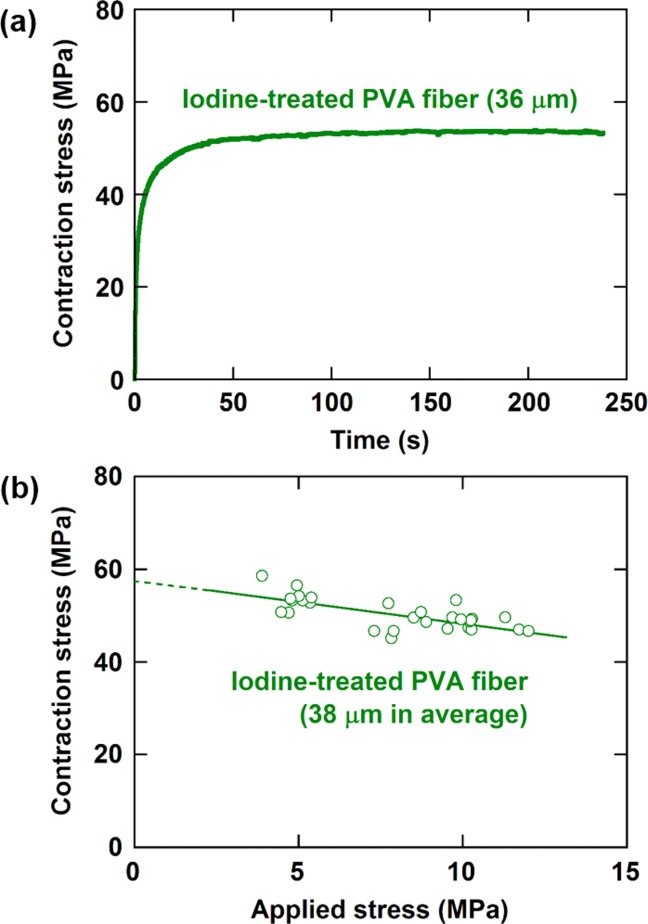
Deswelling behavior of iodine-treated PVA fibers with the indicated diameters. (a) Changes in the contraction stress of the fiber with time after methanol vapor were removed from the atmosphere of the fiber, which had been in equilibrium with saturated methanol vapor. (b) Contraction stress of the fiber at saturation long after methanol vapor was removed from the atmosphere of the fiber, which had been in equilibrium with saturated methanol vapor, plotted against the tensile stress applied during iodine treatment for preventing shrinkage. [Color figure can be viewed in the online issue, which is available at http://wileyonlinelibrary.com.]

When the iodine-treated PVA fiber swollen in saturated methanol vapor is fixed at a constant length and then methanol vapor is removed from the atmosphere, a contraction stress arises due to deswelling. This contraction stress varied with time as shown in Figure 6(a). The contraction stress at saturation long after methanol vapor was removed varied depending on the tensile stress applied during iodine treatment for preventing shrinkage, as shown in Figure 6(b). The contraction stress reached 59 MPa. Swelling and deswelling of the iodine-treated PVA were reversible.

**Figure 7:**
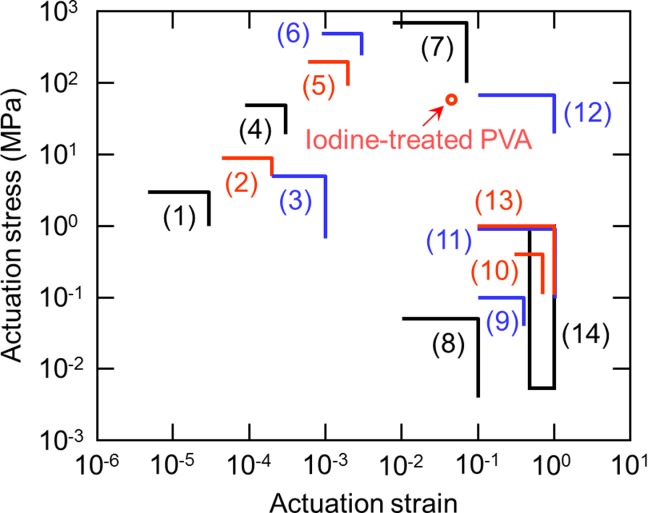
Comparison of the performance of iodine-treated PVA fibers with other actuation systems, including mechanical systems, on a map shown by Kim et al.^19^: (1) low strain piezoelectric, (2) high strain piezoelectric, (3) piezoelectric polymer, (4) thermal expansion (10 K), (5) magnetorictor, (6) thermal expansion (100 K), (7) shape memory alloy, (8) moving coil transducer, (9) solenoid, (10) muscle, (11) pneumatic, (12) hydraulic, (13) polymer gel, and (14) PAN actuator. [Color figure can be viewed in the online issue, which is available at http://wileyonlinelibrary.com.]

The volumetric energy density defined as [contraction stress] × [swelling strain]/2 and the gravimetric energy density defined as [volumetric energy density]/[density] were calculated to be 1.3 × 10^6^ J m^−3^ and 9.6 × 10^2^ J kg^−1^, respectively, using the contraction stress of 59 MPa, the swelling strain of 4.4%, and the density of 1.3 × 10^3^ kg m^−3^. The actuation stress of polymer gels reported to date is up to an order of 1 MPa.^19^ A poly(acrylonitrile) gel fiber, for example, produces a contraction stress of about 2 MPa by changing pH.^20^ The actuation stress of muscles is much lower and is in the range of 0.1–0.4 MPa.^21^ The contraction stress and energy densities of the iodine-treated PVA fiber developed in the current study are much greater than the values so far reported for polymer gels. These values are also greater than the actuation stress of 15 MPa and the gravimetric energy density of 1.6 × 10^2^ J kg^−1^ reported for electroirradiated poly(vinylidene fluoride-trifluoroethylene) copolymer due to a giant electrostriction.^1^ Figure 7 compares the levels of stress and strain generation for the iodine-treated PVA fibers and various actuation systems, including mechanical systems, on a map shown by Kim et al.^19^

The Gibbs free energy change at swelling consists of the energy change due to mixing, Δ*G*_mix_, and that due to strain, Δ*G*_strain_. The swelling takes place if Δ*G*_mix_ is negative and larger in magnitude than Δ*G*_strain_ which takes a positive value. The swelling equilibrium is reached when the chemical potential of the solvent in the swollen polymer is equal to the chemical potential of the solvent outside the polymer. The magnitude of the swelling energy, −(Δ*G*_mix_ + Δ*G*_strain_), at equilibrium gives the maximum available energy for actuation. The energy change Δ*G*_mix_ consists of the entropy gain due to mixing and the enthalpy change due to polymer–solvent interaction. Based on the Flory-Huggins theory, Δ*G*_mix_ is given by the following equation:


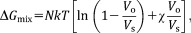
(2)

where *k* is the Boltzmann constant, *χ* is the Flory-Huggins interaction parameter, *T* is the absolute temperature, *N* is the number of the solvent molecules in the swollen polymer, *V*_o_ is the initial volume of the polymer, and *V*_s_ is the volume of the swollen polymer which depends on *N*. For ordinary polymer gels, Δ*G*_strain_ relates to the entropic force of the rubber elasticity, and the degree of swelling is represented by the well-known Flory-Rehner equation. For the iodine-treated PVA, on the other hand, Δ*G*_strain_ relates to the energetic force of glassy polymer as this material is in a glassy state at room temperature. This is one of the most significant differences between the ordinary polymer gels and the iodine-treated PVA. If the elastic modulus, *E*, and the Poisson ratio, *ν*, do not change during the swelling process, Δ*G*_strain_ can be derived as follows:


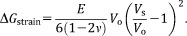
(3)

If *E* and *ν* change during the swelling process, the average of the values for dry and swollen states can be approximately used for these values. The anisotropy should also be taken into account for considering the swelling behavior of fibers. Equation 3 suggests that the swelling energy −(Δ*G*_mix_ + Δ*G*_strain_) decreases as the elastic modulus increases. It is considered, therefore, that the extraordinary large swelling energy of the iodine-treated PVA originates from a large negative value of *χ*. We are planning to determine *χ* for this material by measuring the absorption isotherm.

### Jumping Motion

**Figure 8:**
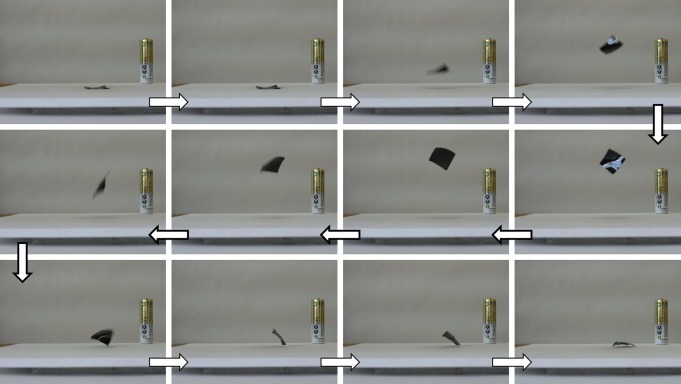
Photographs of the iodine-treated PVA film when it jumped on an alumina plate wiped with a methanol-wetted cloth. Photographs were taken at an interval of 0.04 s after the film was placed on the plate with the convex side upward. An AA-size battery was placed for scale reference. [Color figure can be viewed in the online issue, which is available at http://wileyonlinelibrary.com.]

The iodine-treated PVA films jumped on an alumina plate wiped with a methanol-wetted cloth. On the alumina plate, the film bent into a convex downward shape. By turning the film upside down, it jumped as shown in Figure 8 (see movies in Supporting Information). If the film happened to land with the convex side upward after the first jump, it spontaneously jumped again. The height of the jump reached ∼13 cm for a 2 cm × 2 cm film. The film jumped even when the alumina plate was replaced with a graphite plate or methanol was replaced with acetone and even after the film was heated to at least 200 °C for 30 min.

**Figure 9:**
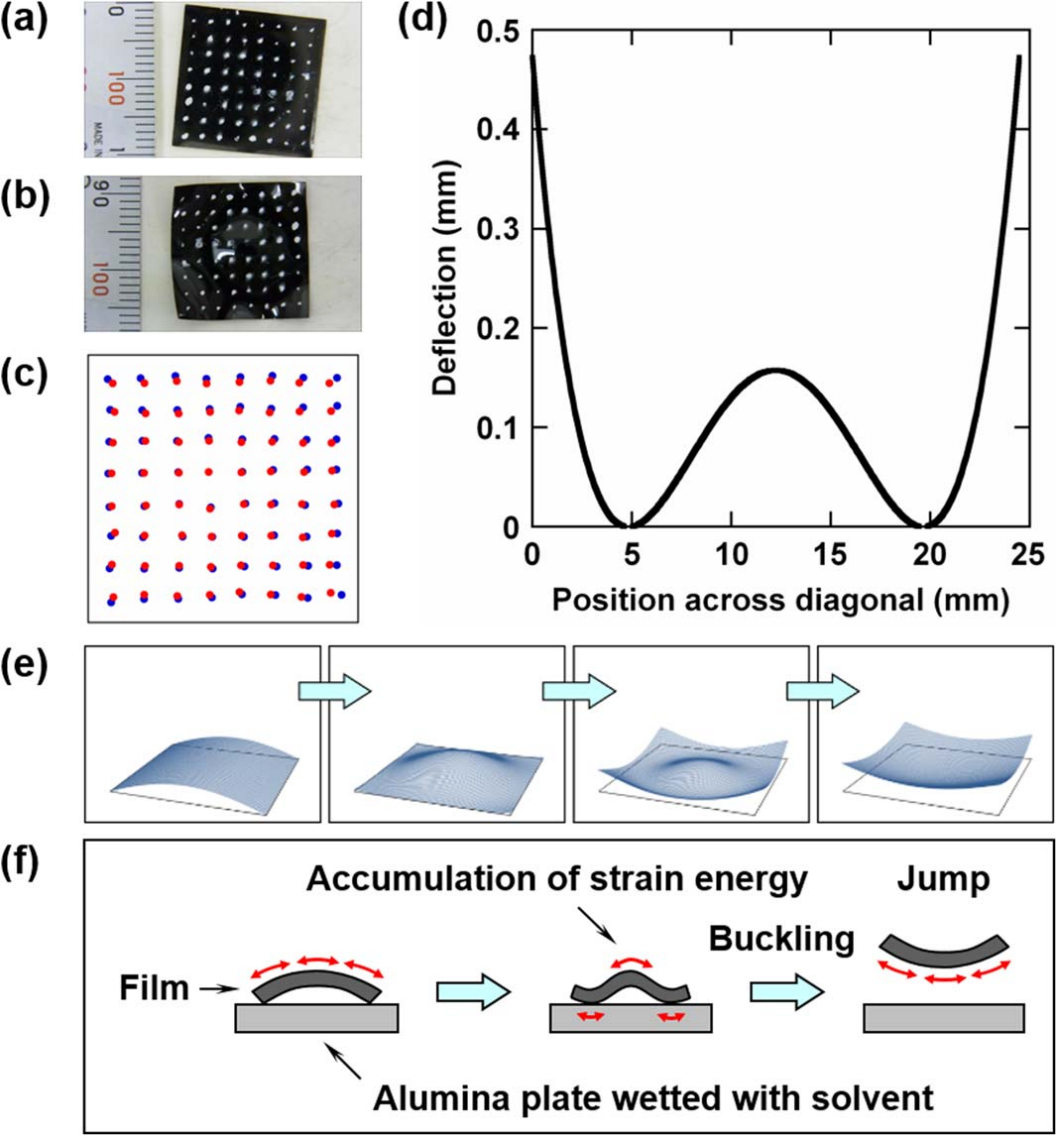
Photographs of white-dot-painted iodine-treated PVA film (a) before deformation and (b) just before jump, respectively. (c) Positions of white dots before deformation (blue circles) and just before jump (red circles). (d) Deflection across a diagonal of iodine-treated PVA film just before jump. Schematic illustrations of iodine-treated PVA film showing (e) changes in shape during jumping motion and (f) mechanism for jumping where arrows represent relative expansion at one side of the film when compared with another side. [Color figure can be viewed in the online issue, which is available at http://wileyonlinelibrary.com.]

To elucidate the mechanism for jumping, an image analysis was performed, which determined the changes in the film shape during jumping motion. The iodine-treated PVA film was painted with white dots and observed with a CCD camera from above as shown in Figure 9(a,b). The displacement of the white dots observed from above shown in Figure 9(c) was primarily caused by the bending of the film as the film was subjected to swelling and deswelling simultaneously at the opposing surfaces, and the length change of the neutral plane (middle surface of the film) was small. The distance between neighboring dots after deformation relative to the distance before deformation gives the cosine of the inclination angle of the film surface at each point. Figure 9(d) shows the deflection across a diagonal of the film just before jump, calculated from the inclination angles.

The bird's-eye and cross-sectional views of the film during jumping motion are schematically shown in Figure 9(e,f). Swelling and deswelling of the film with the solvent vapor occurred at the surface facing the alumina plate and the opposite surface, respectively, and the resulting dilatation and contraction at opposing surfaces caused bending of the film. By turning the bent film upside down with the convex side upward, the film shape began to change from convex upward to convex downward. This change did not proceed gradually in the order of convex upward, flat, and then convex downward shapes but occurred suddenly in a manner of buckling without ever assuming a flat shape. This is because the change from convex upward to convex downward began at the film edges in contact with the alumina plate rather than at the central part of the film, which prevented the film from assuming a flat shape. The central part of the film slapped the alumina plate when this part changed from convex upward to convex downward and the film jumped due to the reaction force.

For the film to jump, the strain energy needs to be accumulated until it reaches to the amount sufficient for jump. The film jumps when it buckles as shown in Figure 9(f). The Euler equation for buckling suggests that the critical stress at which buckling takes place increases with the elastic modulus. A high elastic modulus of the iodine-treated PVA film retards the commencement of buckling and contributes to providing a long time to accumulate the strain energy produced by swelling and deswelling.

The strain energy is converted into the kinetic energy through buckling, which is accompanied by an energy dissipation due to viscous character for the viscoelastic material. This energy dissipation decreases as the relaxation time increases. A long relaxation time of the iodine-treated PVA contributes to suppressing the energy dissipation.

Various methods have been developed to produce bending, twisting, and folding motions of polymer films based on the spatial heterogeneities within the films such as multilayered structure, gradient of crosslinking, and heterogeneity of the porosity.^22^–^24^ In contrast, the jumping motion of the iodine-treated PVA film is caused not by the heterogeneity of the degree of iodine treatment but by the swelling and deswelling at opposing surfaces of the film. The iodine-treated PVA film is made capable of jumping relying on the combination of a large swelling energy, a high elastic modulus, and a long relaxation time.

## Conclusions

We have developed an iodine-treated PVA showing an extraordinarily large deswelling stress (59 MPa), extraordinarily large volumetric and gravimetric energy densities (1.3 × 10^6^ J m^−3^ and 9.6 × 10^2^ J kg^−1^, respectively), a high elastic modulus (a few GPa), a long relaxation time, and an excellent thermal stability when compared with ordinary polymer gels. The performance of this material can be demonstrated by a jump of a film. The current findings support the potential to create a new class of materials with advantageous properties that ordinary polymer gels do not possess.

## References

[1] Zhang QM, Bharti V, Zhao X (1998). Science.

[2] Kuhn W, Hargitay B, Katchalsky A, Eisenberg H (1950). Nature.

[3] Steinberg IZ, Oplatka A, Katchalsky A (1966). Nature.

[4] Baughman RH, Shacklette LW, Elsenbaumer RL, Plichta E, Becht C, Bredas JL, Chance RR (1990). Conjugated Polymeric Materials: Opportunities in Electronics, Optoelectronics, and Molecular Electronics.

[5] Osada Y, Okuzaki H, Hori H (1992). Nature.

[6] Okuzaki H, Funasaka K (1996). J. Polym. Sci. Part B: Polym. Phys.

[7] Yu Y, Nakano M, Ikeda T (2003). Nature.

[8] Hosono N, Furukawa H, Masubuchi Y, Watanabe T, Horie K (2007). Colloid Surface B.

[9] Xia H, Takasaki M, Hirai T (2010). Sens. Actuators A.

[10] Lima MD, Li N, de Andrade MJ, Fang S, Oh J, Spinks GM, Kozlov ME, Haines CS, Suh D, Foroughi J, Kim SJ, Chen Y, Ware T, Shin MK, Machado LD, Fonseca AF, Madden JDW, Voit WE, Galvão DS, Baughman RH (2012). Science.

[11] Chun K, Kim SH, Shin MK, Kwon CH, Park J, Kim YT, Spinks GM, Lima MD, Haines CS, Baughman RH, Kim SJ (2014). Nat. Commun.

[12] Choi Y, Miyasaka K (1993). J. Appl. Polym. Sci.

[13] Uddin AJ, Narusawa T, Gotoh Y (2011). Polym. Eng. Sci.

[14] Igarashi T, Shioya M, Yamashita J (2003). Proc. Annu. Meet. Soc. Fib. Sci. Technol. Japan.

[15] Bin Y, Chen Q, Nakamura Y, Tsuda K, Matsuo M (2007). Carbon.

[16] Sashio M, Tanaka M (1985). J. Polym. Sci. Polym. Chem. Ed.

[17] Igarashi T (2004).

[18] Bunn CW (1948). Nature.

[19] Kim C, Kim KJ (2006). Sens. Actuators A.

[20] Umemoto S, Okui N, Sakai T, DeRossi D, Kajiwara K, Osada Y, Yamauchi A (1990). In Polymer Gels.

[21] Huber JE, Fleck NA, Ashby MF (1997). Proc. R. Soc. Lond. A.

[22] Jeong KU, Jang JH, Kim DY, Nah C, Lee JH, Lee MH, Sun HJ, Wang CL, Chengc SZD, Thomas EL (2011). J. Mater. Chem.

[23] Gracias DH (2013). Curr. Opin. Chem. Eng.

[24] Zhang Y, Ionov L (2014). ACS Appl. Mater. Interfaces.

